# *DPH1* Gene Mutations Identify a Candidate SAM Pocket in Radical Enzyme Dph1•Dph2 for Diphthamide Synthesis on EF2

**DOI:** 10.3390/biom13111655

**Published:** 2023-11-16

**Authors:** Koray Ütkür, Sarina Schmidt, Klaus Mayer, Roland Klassen, Ulrich Brinkmann, Raffael Schaffrath

**Affiliations:** 1 Institut für Biologie, Fachgebiet Mikrobiologie, Universität Kassel, 34132 Kassel, Germany; k.uetkuer@uni-kassel.de (K.Ü.); sarina.schmidt1@t-online.de (S.S.); roland.klassen@uni-kassel.de (R.K.); 2 Roche Pharma Research and Early Development (pRED), Large Molecule Research, Roche Innovation Center Munich, 82377 Penzberg, Germany; klaus.mayer.km1@roche.com (K.M.); ulrich.brinkmann@roche.com (U.B.)

**Keywords:** *Saccharomyces cerevisiae*, SAM, radical SAM enzymes, EF2 diphthamide modification, Dph1•Dph2, diphtheria toxin, ADP ribosylation

## Abstract

In eukaryotes, the Dph1•Dph2 dimer is a non-canonical radical SAM enzyme. Using iron-sulfur (FeS) clusters, it cleaves the cosubstrate S-adenosyl-methionine (SAM) to form a 3-amino-3-carboxy-propyl (ACP) radical for the synthesis of diphthamide. The latter decorates a histidine residue on elongation factor 2 (EF2) conserved from archaea to yeast and humans and is important for accurate mRNA translation and protein synthesis. Guided by evidence from archaeal orthologues, we searched for a putative SAM-binding pocket in Dph1•Dph2 from *Saccharomyces cerevisiae*. We predict an SAM-binding pocket near the FeS cluster domain that is conserved across eukaryotes in Dph1 but not Dph2. Site-directed *DPH1* mutagenesis and functional characterization through assay diagnostics for the loss of diphthamide reveal that the SAM pocket is essential for synthesis of the décor on EF2 in vivo. Further evidence from structural modeling suggests particularly critical residues close to the methionine moiety of SAM. Presumably, they facilitate a geometry specific for SAM cleavage and ACP radical formation that distinguishes Dph1•Dph2 from classical radical SAM enzymes, which generate canonical 5′-deoxyadenosyl (dAdo) radicals.

## 1. Introduction

In all domains of life, radical SAM (RS) enzymes use iron-sulfur (FeS) clusters together with S-adenosyl-methionine (SAM) as a cosubstrate for biocatalysis [1,2]. Members of the classical RS enzyme family possess a SAM motif (-CX_3_CX_2_C-), with each cysteine coordinating one iron of a cubic 4Fe-4S cluster [3]. The fourth unique iron binds SAM to set the catalytic framework of a site-differentiated cluster for electron transfer and reductive SAM cleavage. As a result, 5′-deoxyadenosyl (dAdo) radicals are formed for use in biosynthetic reactions, including but not limited to chemical decorations of biological macromolecules (i.e., lipids, proteins and nucleic acids) [4,5].

Considered ‘radically’ different from the classical RS family [6] are two related enzymes conserved among archaea (Dph2•Dph2) and eukaryotes including yeast, plants and humans (Dph1•Dph2) [7,8,9,10]. They incorporate FeS clusters and bind SAM [11,12], but rather than producing dAdo radicals, they generate from SAM a non-canonical 3-amino-3-carboxy-propyl (ACP) radical for prime modification of translation elongation factor 2 (EF2) with diphthamide [13,14]. The décor can be attacked for ADP ribosylation and inactivation of EF2 by diphtheria toxin (DT), a causative agent of the human diphtheria disease (hence its name) [15,16,17]. Diphthamide is formed in four steps encoded by a complex gene (*DPH1*–*DPH8*) network [18,19,20] and supports EF2 accuracy during mRNA translation and protein synthesis. Hence, the loss of diphthamide can trigger mistranslation and frameshift errors with negative consequences for proteostasis, cell proliferation and development [21,22,23,24].

The latter notion is particularly manifested by traits linked with *DPH* gene mutations in animal or human cells and collectively known as diphthamide deficiency syndrome (DDS) [10,25,26,27]. Prominent DDS phenotypes include developmental traits such as embryonic lethality in *DPH* gene-knockout mice [28,29] or craniofacial and neurodegenerative features observed in humans with *DPH1*, *DPH2* or *DPH5* gene mutations [25,26,27,28,29,30]. Molecularly, DDS underlies reduced activity of diphthamide synthesis enzymes including DPH1•DPH2, which upon mutation can block ACP formation and thus fail to initiate the diphthamide pathway [8,10,11,14]. Taking the emerging diphthamide relevance for DDS into account, we further studied the ACP formation step using yeast Dph1•Dph2 as a non-canonical RS enzyme model. Sequence alignments and structure modeling with *Cmn*Dph2•Dph2, an orthologue of the yeast dimer from the archaeon *Candidatus methanoperedens nitroreducens* [14], were guided into site-directed yeast *DPH1* gene mutagenesis. In this way, we identified candidate residues for a SAM pocket, which we show is essential for diphthamide synthesis on EF2, presumably by conferring SAM binding to the yeast RS enzyme Dph1•Dph2.

## 2. Materials and Methods

### 2.1. Strains, Media, Growth Conditions and Assays

The *S*. *cerevisiae* strains used or generated throughout this study are listed in Table S1. BY4741-derived yeast strains carrying site-specific substitution or deletion mutations at the *DPH1* chromosomal locus were generated using PCR-mediated protocols, oligonucleotides, gene-specific primers (Table S2) and plasmid templates as previously described [10,19,31]. HA epitope tagging at the *DPH1* wild-type and mutant loci for immunological Dph1 gene product detection involved previously described PCR-methods [32]. For genomic manipulations, the DNA transformations utilized a standard lithium-acetate protocol [33]. The strains were grown in complete yeast peptone dextrose (YPD) or minimal synthetic defined (SD) media [34] at 30 °C unless otherwise stated. For antifungal response assays, 10-fold serial cell dilutions of *S. cerevisiae* tester strains (starting OD_600_: 1.5) were spotted onto YPD plates lacking or containing 5–15 μg/mL sordarin (Sigma-Aldrich, St. Louis, MO, USA). Incubation took place for 2–4 days at 30 °C. The growth assays in response to diphtheria toxin (DT) fragment A involved transformation with single-copy vector pSU9 [19] for galactose-inducible expression of the cytotoxic ADP-ribosylase activity as previously described [10,21].

### 2.2. Sequence Alignments and Protein Modeling Based on the Archaeal CmnDph2 Structure

The amino acid sequence of *Cmn*Dph2 (UniProt-ID A0A062UZ78) was aligned to Dph1 and Dph2 from *S. cerevisiae* (UniProt-IDs P40487 and P32461), *A. thaliana* (UniProt-IDs Q8RWW3 and A0A1I9LRW3), *D. melanogaster* (UniProt-IDs Q9VTM2 and Q9VFE9), *M. musculus* (UniProt-IDs Q5NCQ5 and Q9CR25) or *H. sapiens* (UniProt-IDs Q9BZG8 and Q9BQC3) with ClustalOmega (https://www.ebi.ac.uk/Tools/msa/clustalo/ accessed on 11 October 2023) and illustrated with Jalview (https://www.jalview.org/development/archive/Version-2_11_2_7/ accessed on 11 October 2023). The structure of *Cmn*Dph2 in complex with SAM (PDB:6BXN) was solved [14] and guided into Dph1•Dph2 models using AlphaFold and ColabFold platforms [35,36] with yeast Dph1 and Dph2 protein sequences (UniProt-IDs as shown above) as recently described [10] (https://colab.research.google.com/github/sokrypton/ColabFold/blob/main/AlphaFold2.ipynb accessed on 11 October 2023). Visualization of the structures used PyMOL (https://pymol.informer.com/1.3/ accessed on 11 October 2023), and the candidate SAM pocket residues were curated manually.

### 2.3. Assaying Diphthamide-Modified EF2 and ADP Ribosylation (ADPR) of EF2 with DT

Diagnosis of the EF2 diphthamide modification states in vivo involved Western blots of the total yeast cell extracts and antibodies that detect global EF2 pools irrespective of diphthamide modification (anti-EF2[pan]) or specifically recognize unmodified forms of EF2 (*anti-EF2[no diphthamide]*) [37]. Both antibodies were originally raised to detect human EF2 [37]. As the diphthamide contexts in human (708-TLHADAIHRGGGQIIPT-724) and yeast (692-TLHADAIHRGGGQIIPT-708) cells are identical, *anti-EF2(no diphthamide)* is also suited to differentiating the diphthamide modification states of EF2 from *S. cerevisiae* [21]. The quantification of unmodified EF2 signals relative to *dph1*∆ was densitometrically analyzed using ImageJ version 1.50i.

The total yeast cell extracts were generated as previously described [38], and the protein concentrations were determined with the Bradford assay [39]. The Lämmli samples were run by SDS-PAGE (12% (*w*/*v*) polyacrylamide) and blotted onto PVDF membranes (Millipore, Burlington, MA, USA). These were probed overnight at 4 °C with the *anti-EF2(pan)* and *anti-EF2(no diphthamide)* antibodies [21] and developed with anti-rabbit secondary antibody HRP conjugate (Dianova, Hamburg, Germnamy; working concentration = 1:5000) and Lumi-Light Western blotting substrate (Roche, Basel, Switzerland) as described previously [21,37]. Protein loading was controlled in parallel Western blots with anti-Cdc19 antibodies kindly donated by Dr. Jeremy Thorner (University of California, Berkeley, CA, USA) and recognized yeast pyruvate kinase (i.e., Cdc19). Similarly, anti-HA Western blots (Invitrogen, Waltham, MA, USA, 2-2.2.14) were performed to verify the expression of HA-tagged Dph1 gene products. Diphthamide-dependent ADPR acceptor activity from EF2 in the presence of DT was tested in vitro [40] using the total yeast extracts and biotinylated NAD^+^ as ADP-ribosyl donors for the DT reaction essentially as previously described with human and yeast EF2 resources [10,27,40].

## 3. Results and Discussion

### 3.1. In Search for a Potential SAM Pocket in the Yeast Dph1•Dph2 Heterodimer

**Figure 1:**
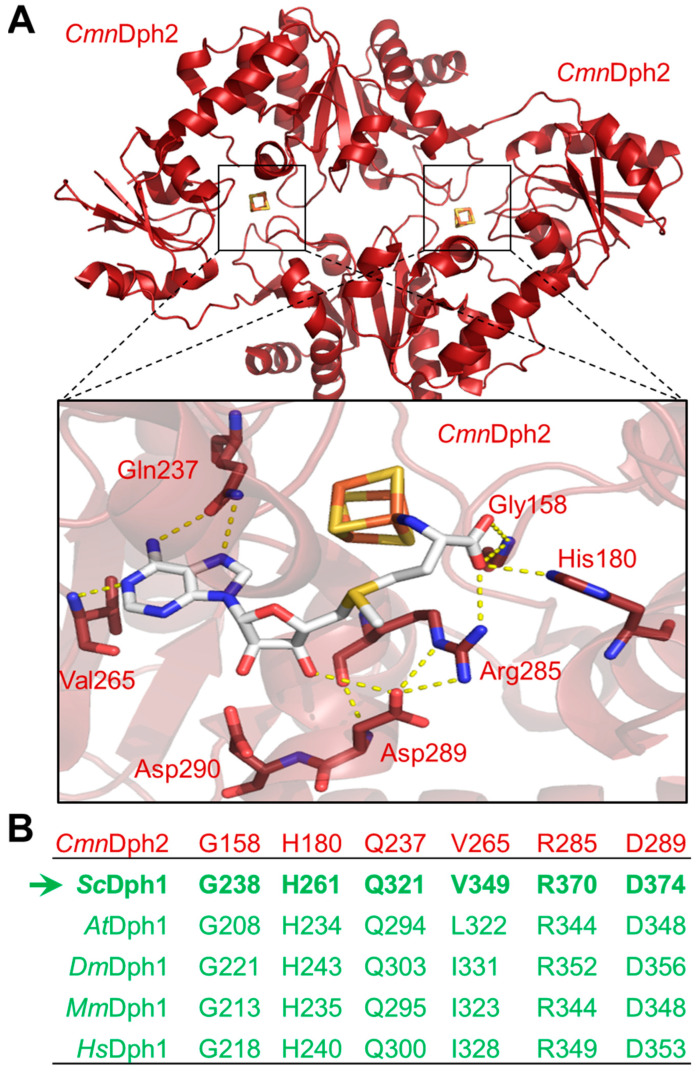
The SAM-binding pocket in archaeal Dph2 subunits of ACP synthase dimers from *Candidatus methanoperedens nitroreducens* (*Cmn*Dph2•Dph2) was conserved in the Dph1 subunit of eukaryal counterparts (Dph1•Dph2). (**A**) Crystal structure of *Cmn*Dph2•Dph2 homodimer in complex with SAM (PDB: 6BXN) revealed specific SAM-interacting residues (orange dotted lines represent polar contacts): Gly-158, His-180, Gln-237, Val-265, Arg-285, Asp2-89 and Asp-290 [14]. (**B**) Excerpt of the amino acid sequence alignment between *Cmn*Dph2 (in red) and Dph1 subunits from model eukaryotes (in green): *S. cerevisiae* (*Sc*), *A. thaliana* (*At*), *D. melanogaster* (*Dm*), *M. musculus* (*Mm*) and *H. sapiens* (*Hs*). The table sums up eukaryal Dph1 residues that were conserved or similar to the SAM pocket from *Cmn*Dph2. Residues identical between *Cmn*Dph2 and *Sc*Dph1 are labeled in bold, with the arrow highlighting the amino acid positions chosen for alanine-specific substitution mutagenesis of yeast gene *DPH1*. For full alignment details, see Figures S1 and S2.

Structural studies with prokaryotic orthologues of the yeast Dph1•Dph2 heterodimer showed the presence of FeS clusters in each subunit of the Dph2•Dph2 homodimer from the archaea *Pyrococcus horikoshii* (*Ph*) [7,11], *Candidatus methanoperedens nitroreducens* (*Cmn*) [14] and *Methanobrevibacter smithii* (*Ms*) [41]. In addition, crystallization of the *Cmn*Dph2•Dph2 dimer in complex with SAM demonstrated [14] a subset of amino acid residues (Figure 1A) surrounding the FeS and SAM cofactors (i.e., Gly-158, His-180, Gln-237; Val-265, Arg-285, Asp-289, Asp-290). Although intramolecular contacts to SAM are suggestive for a potential role of these residues in SAM coordination (Figure 1A), there is hardly evidence in support of their catalytic relevance except for Arg-291 from *Ms*Dph2 (equivalent to Arg-289 in *Ph*Dph2 and Arg-285 in *Cmn*Dph2 (Figure 1A)), which is proposed to guide the ACP radical to react with EF2 [41].

Therefore, we examined whether the residues of interest were invariant between archaeal and eukaryotic enzymes including the Dph1•Dph2 dimer from yeast. In the search for a potential SAM-binding pocket that is conserved among eukarya, the amino acid sequence of *Cmn*Dph2 was aligned to both Dph1 and Dph2 from *Saccharomyces cerevisiae* (*Sc*), *Arabidopsis thaliana* (*At*), *Drosophila melanogaster* (*Dm*), *Mus musculus* (*Mm*) and *Homo sapiens* (*Hs*) (Figure S1). Except for one residue (i.e., Asp-290), all others appeared to be invariant across all species in Dph1 but strikingly not in the eukaryal Dph2 subunit (Figure 1B and Figure S2). *Cmn*Dph2 Val-265 is considered to be conserved since Dph1 sequences from the eukaryotes above contain either a valine residue (*Sc*Dph1 Val-349) or instead a branched chain amino acid (e.g., leucine or isoleucine) at the position of interest (i.e., *At*Dph1 Leu-349, *Dm*Dph1 Ile-331, *Mm*Dph1 Ile-323 and *Hs*Dph1 Ile-328) (Figure 1B). Of note, in contrast to the Dph1 subunits, no eukaryal Dph2 query sequences showed overall high degrees of conservation (Figure S2). Thus, only a few rare, randomly scattered residues were found (i.e., *At*Dph2 Asp-342 as well as *Dm*Dph2 Ile-309 and Asp-335 (Figure S2)) to be similar between the archaeal and eukaryal Dph2 sequences.

Using AlphaFold2-based models of the *Cmn*Dph2•Dph2 dimer, we next aligned the conserved Dph1 residues (i.e., Gly-238, His-261, Gln-321, Val-349, Arg-370 and Asp-374 (Figure 1)) to the SAM-bound structure of *Cmn*Dph2 [14]. In line with the SAM pocket in *Cmn*Dph2 near the 4Fe-4S cluster [14], all the conserved Dph1 residues were found to be positioned around the two cofactors too. As for the rare residues scattered among a few eukaryal Dph2 sequences excluding yeast (Figure S2) and similar to *Cmn*Dph2, our models would not suggest a putative SAM pocket in the Dph2 subunit of the heterodimer.

In conclusion, evidence from our sequence alignments supports the idea of a SAM pocket in the yeast Dph1•Dph2 heterodimer and is possibly related to the archaeal Dph2•Dph2 homodimer (Figure 1). Moreover, this potential SAM-binding pocket is exclusively conserved in Dph1 subunits from eukaryotes and not from Dph2. This raises the intriguing question as to whether, and if so, how such asymmetry in the ability to bind SAM between the two FeS cluster-carrying subunits contributes to catalysis of the dimeric RS complex. In a previous report by Dong et al. in 2019, the two FeS clusters were proposed to differ due to catalytic versus accessory or regulatory functions [8].

### 3.2. Conserved Residues in Dph1 Qualify for a SAM Pocket Relevant to Diphthamide Synthesis

**Figure 2:**
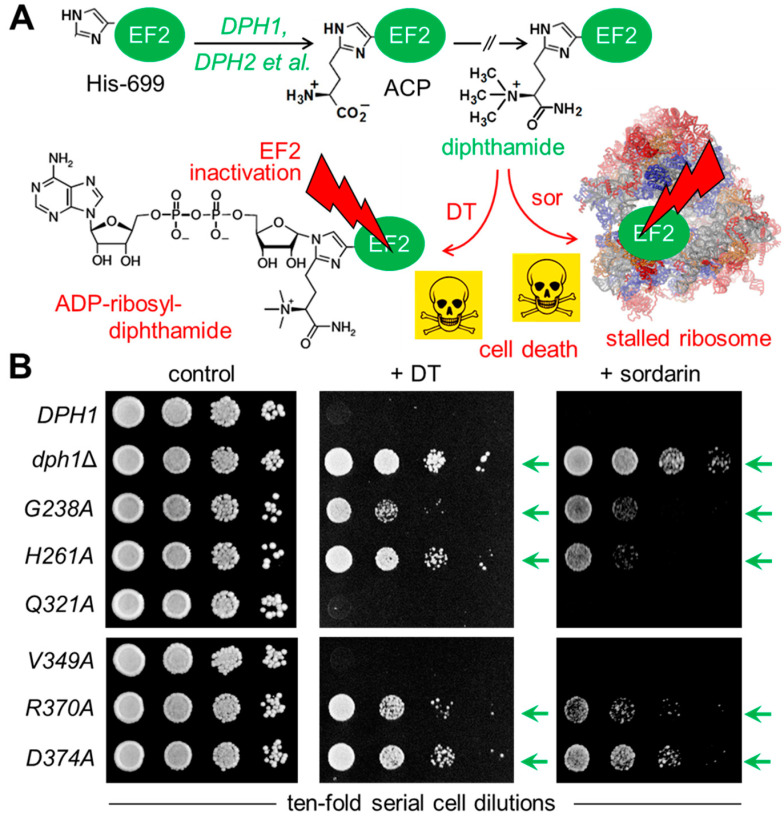
Site-directed *DPH1* mutagenesis uncovered a candidate SAM pocket in yeast Dph1•Dph2 relevant to diphthamide synthesis on EF2. (**A**) Simplified scheme showing diphthamide synthesis initiated by the *DPH1* and *DPH2* gene products and other factors (et al.) to modify His-699 on yeast EF2 with ACP. Subsequent enzymatic steps that completed diphthamide are not shown in detail for simplicity. Diphthamide is pathologically relevant; it can be hijacked by diphtheria toxin (DT) for inhibitory ADP ribosylation of EF2 and induction of cell death (skull and crossbones) or complexed by antifungal sordarin (sor) to irreversibly stall elongating ribosomes. (**B**) Cell growth assays in response to DT and sordarin to phenotypically diagnose diphthamide synthesis on EF2. As indicated, yeast tester strains comprised wild-type (*DPH1*) and null mutant (*dph1*∆) controls as well as the candidate SAM pocket mutants (*G238A*, *H261A*, *Q321A V349A*, *R370A* and *D374A*). Ten-fold serial cell dilutions were cultivated for 2–3 days at 30 °C without DT or sordarin (left panel: control) under conditions of endogenous DT fragment A production from a galactose-inducible expression plasmid (middle panel: + DT) [19] or in the presence of 12.5 µg/mL antifungal (right panel: +sordarin) sufficient to inhibit the wild-type (*DPH1*) control. Green arrows indicate DT and sordarin resistance.

To examine the relevance of the conserved Dph1 residues, site-directed substitutions were generated at the chromosomal *DPH1* locus using PCR-based engineering that allowed for protein expression under native promoter control. In tandem with parallel HA epitope tagging and Western blots, we detected the mutant Dph1 proteins (*G238A*, *H261A*, *Q321A*, *V349A*, *R370A* and *D374A*) at expression levels which were comparable to a wild-type (*DPH1*) reference (Figure S3) and much less affected than *C368S*, a highly labile catalytic mutant [8] used as a negative control. Next, their diphthamide synthesis capacities were investigated in vivo with assays that monitored the diphthamide-dependent inhibition of EF2 and cell growth by DT and the antifungal sordarin (Figure 2A).

First, the mutant collection was transformed with a plasmid (pSU9) allowing for cytotoxic expression of the catalytic DT subunit under *GAL1* promoter control [19,21]. Upon cultivation under inducing the conditions (i.e., with galactose added to the growth medium as the sole carbon source (Figure 2B)), a sensitive DT phenotype leading to cell death was seen with the diphthamide-proficient wild-type (*DPH1*) strain (Figure 2B). In contrast, significant DT resistance traits were typical of the diphthamide-deficient *DPH1* deletion strain (*dph1*Δ) used as a negative control and in association with the *G238A*, *H261A*, *R370A* and *D374A* substitution mutants (Figure 2B). Intriguingly, both the *Q321A* and *V349A* mutants appeared to express a DT-sensitive phenotype (Figure 2B) similar to that of the wild type. To complement our DT data and further analyze the ability to produce diphthamide, the mutant collection was assayed with sordarin (Figure 2B), an antifungal that kills yeast by inhibiting EF2 in a fashion dependent on diphthamide but different from DT’s mode of action [42,43,44]. The sordarin phenotype of the *D374A* mutant and the diphthamide-deficient *dph1*∆ control, when compared to each other, showed robust resistance against growth inhibition by the antifungal (Figure 2B). In addition, the substitution mutants *G238A*, *H261A* and *R370A* showed protection against toxic sordarin doses too, yet this was to a lesser degree than either *D374A* or the *dph1*∆ control (Figure 2B). The two other mutants (*Q321A* and *V349A*), which was found to be DT-sensitive earlier, also appeared unchanged from the wild-type *DPH1* cells with regard to their sordarin responses (Figure 2B).

As shown previously, resistances toward DT and sordarin are bona fide phenotype diagnostics for failure to initiate or complete diphthamide synthesis on EF2 in yeast cells [10,19,21,43,44]. Thus, four out of the six *DPH1* substitutions tested apparently caused defects that are characteristic for a diphthamide loss-of-function (*dph1*Δ) mutant. This confirms that the majority of the candidate Dph1 residues targeted for mutagenesis are functionally relevant and play important roles in vivo for the Dph1•Dph2 enzyme to decorate EF2 with diphthamide in yeast.

### 3.3. Unmodified EF2 from the SAM Pocket Mutants Escapes ADP Ribosylation by DT

**Figure 3:**
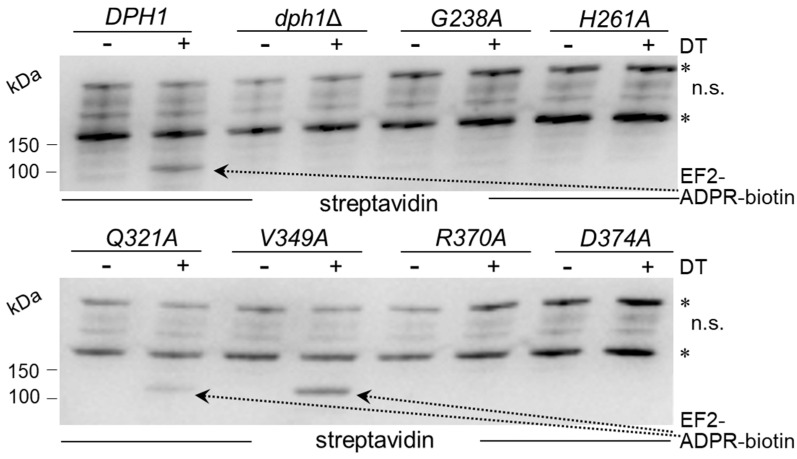
Diphthamide-dependent ADP ribosylation of EF2 by DT in vitro. Total protein extracts obtained from yeast strains with the indicated genetic backgrounds were treated with (+) or without (−) DT (200 ng) in the presence of 5 µM biotin-NAD at 25 °C for 1 h. Detection of the biotin moiety transferred with ADP-ribose to EF2 by DT involved Western blots with a streptavidin peroxidase conjugate. The reaction product (EF2-ADPR-biotin) with a molecular weight of ~100 kDa is denoted by dotted arrows. Unspecific (n.s.) bands marked with an asterisk likely represent endogenously biotinylated yeast proteins. Note that *V349A* produces wild-type-like EF2-ADPR-biotin signals diagnostic for EF2 diphthamide modification, while significantly weaker signals from *Q321A* suggest reduced (but not entirely abolished) Dph1 activity. Original images can be found in Supplementary Materials (Figure S8).

We next examined directly whether EF2 from the candidate SAM pocket mutants could be hijacked in a diphtamide-dependent fashion by DT for ADP ribosylation. To accomplish this, we subjected the total protein extracts from the various substitution mutants to assays that can monitor ADP ribosylation (ADPR) of EF2 by DT in vitro, a reaction known to be strictly dependent on the diphthamide modification. The protocol used DT, biotinylated NAD^+^ as an ADP-ribosyl donor, EF2 extracts as ADPR acceptors and an HRP-streptavidin conjugate to detect biotin in the ADPR reaction product [10,27,40]. EF2 from the wild-type, *V349A* and *Q321A* extracts were ADP ribosylated by DT, with significantly weak signals seen in the *Q321A* material (Figure 3). These are read-outs diagnostic for wild-type-like diphthamide amounts present on EF2 from *V349A* cells and reduced (but not entirely abolished) diphtamide-modified EF2 amounts in the *Q321A* mutant [10,27,40]. In contrast, EF2 from the other SAM pocket mutants (*G238A*, *H261A*, *R370A* and *D374*) and *dph1*Δ cells lacked detectable ADPR acceptor activity, indicating a loss of diphthamide on EF2 and evasion of the DT attack in these backgrounds (Figure 3).

**Figure 4:**
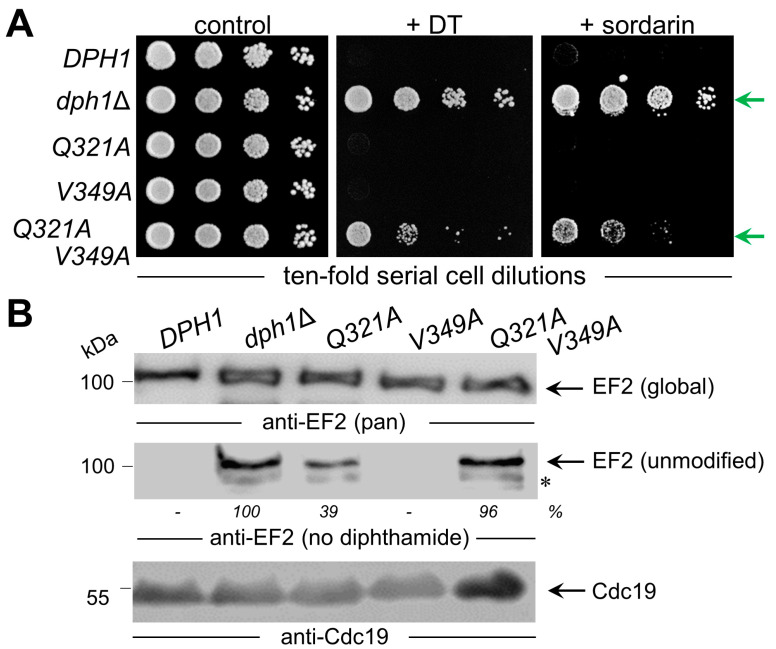
Characterization of single *Q321A* and *V391A* mutants alone and in tandem. (**A**) Phenotypic spot assays diagnostic for diphthamide modification defects. As indicated, the tester strains comprised wild-type (*DPH1*) and null-mutant (*dph1*∆) controls as well as single (*Q321A* or *V391A*) and double (*Q321A V391A*) mutants. Ten-fold serial cell dilutions were cultivated and grown under conditions essentially described in Figure 2B legend. DT and sordarin resistance traits are indicated (green arrows). (**B**) Analysis of the EF2 modification state using *anti-EF2(pan)* antibodies for global EF2 recognition and *anti-eEF2(no diphthamide)* antibodies to specifically detect unmodified EF2 [37]. Quantifications of unmodified EF2 signals relative to *dph1*∆ are given as percentage (%) values. For Western blots, protein extracts were obtained from tester strains with the genetic backgrounds as indicated in (**A**). EF2 degradation products are marked with an asterisk. Detection of Cdc19 with *anti-Cdc19* antibodies is shown in parallel Western blots. Note that the combination of the two single *Q321A* and *V391A* mutations in the double mutant is phenotypically additive (**A**) and causes diphthamide modification defects (**B**) comparable to the null mutant (*dph1*∆). Original images can be found in Supplementary Materials (Figure S9).

The fact that the *Q321A* mutant produced weak yet detectable ADPR signals on EF2 in the presence of DT (Figure 3) suggests residual diphthamide modification and may in part be accountable for the DT- and sordarin-sensitive phenotypes observed in vivo (Figure 2B). In an effort to further characterize the seemingly less important but conserved residues (Gln-321 and Val-349; Figure 1) and understand their contribution, if any, to catalysis by Dph1•Dph2, we probed for potential genetic interaction between the two SAM pocket sites. Upon comparing each single mutant (*Q321A* or *V349A*) alone with the double carrying both mutations (*Q321A* and *V349A*), we found that the two together were phenotypically additive and enhanced the sordarin and DT resistance traits (Figure 4A). Since these phenotypes were otherwise not elicited in each mutant alone, the readout implies that in Dph1, the Gln-321 and Val-349 residues may back up each other in a function so that their combined loss is negative, adding up to the phenotype of each mutant alone (Figure 4A).

Next, we examined the capacity of the single and double mutants to initiate step one of the diphthamide pathway (i.e., the formation of ACP-modified EF2 (Figure 2A)). To accomplish this, we subjected total protein extracts from the control strains (*DPH1* and *dph1*Δ) and the mutants (*Q321A*, *V349A* and *Q321A V349A*) to Western blots using antibodies (i.e., *anti-EF2(no diphthamide)* and *anti-EF2(pan)* (Figure 4B)) which were previously shown to distinguish unmodified EF2 from global EF2 amounts [10,21,37]. Except for the single *V349A* mutant, which accumulated diphthamide-modified EF2 pools similar to the wild type (*DPH1*), the single *Q321A* and double *Q321A V349A* mutants produced immune-responsive signals diagnostic for unmodified EF2 and a defect in the diphthamide pathway (Figure 4B). As with the ADPR assays above (Figure 3), the EF2 modification defect of the single *Q321A* mutant (39%) was weaker than the robust signal of the *dph1*Δ null mutant (Figure 4B). In contrast, the double mutant *Q321A V349A* displayed a pronouced EF2 modification defect (96%) indistinguishable from the *dph1*Δ control (Figure 4B).

**Figure 5:**
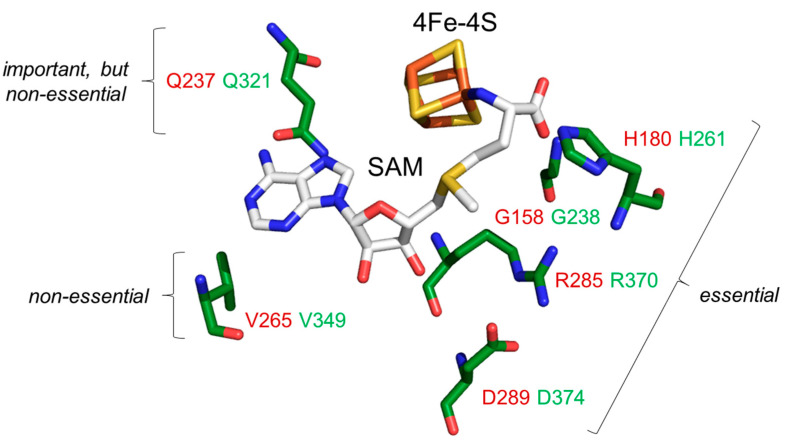
Functional distinctions of individual SAM pocket residues identified in subunit Dph1 from the yeast Dph1•Dph2 dimer. An AlphaFold2-based structural model [35,36] of Dph1 was aligned to the structure of *Cmn*Dph2 in complex with SAM (PDB: 6BXN). Protein structures of *Cmn*Dph2 are hidden, while the 4Fe-4S and SAM cofactors remain shown as sticks. Dph1 amino acids (green) are part of a SAM pocket (Gly-238, His-261, Gln-321, Val-349, Arg-370 and Asp-374 conserved to *Cmn*Dph2 residues (red) with Gly-158, His-180, Gln-237, Val-265, Arg-285 and Asp-289, respectively (see also Figure 1A)). Residues in proximity to the SAM methionine moiety are essential (Gly-238, His-261, Arg-370 and Asp-374), while amino acids close to the adenine are important but nonessential (Gln-321) or dispensable and nonessential (Val-349).

These findings together with data from the genetic and phenotypic interaction assays above (Figure 4A) strongly suggest that interaction between the residues Gln-321 and Val-349, which map side by side in the SAM pocket (Figure 5), is required for full catalytic capacity of the Dph1•Dph2 enzyme. In line with this notion, which implies that SAM pocket residues in Dph1 can be heterogenous and classified according to their functionality (Figure 5), we found that mutation of the other conserved residues (i.e., Gly-238, His-261, Arg-370 and Asp-374) abolished diphthamide synthesis on EF2. Thus, in correspondence to their DT and sordarin resistance phenotypes in vivo (Figure 2B and Figure 4A) and their lack of EF2-based ADPR acceptor activity in vitro (Figure 3), the *G238A*, *R370A*, *H261A* and *D374A* mutants produced robust *anti-EF2(no diphthamide)* Western signals (Figure S4). These are defects deemed essential for Dph1•Dph2 enzyme activity in vivo in typical bona fide *dph1*Δ (or *dph2*Δ) diphthamide mutants qualifyng the Dph1 residues Gly-238, His-261, Arg-370 and Asp-374 (Figure 5).

In sum, our work demonstrates that EF2 produced from the single and double substitution mutants is not or cannot be properly modified by diphthamide. This leads us to conclude that the integrity of residues Gly-238, His-261, Gln-321, Arg-370, Asp-374 and possibly Val-349 (in tandem with Gln-321) is catalytically vital (Figure 5). Overall, the effects of each alanine mutation in the candidate SAM pocket residues are in line with the predictions from the structural models (Figure S5). It is highly likely that the Dph1 residues identified contribute to SAM binding and cleavage by Dph1•Dph2 in order to initiate the modification pathway (Figure 2A).

## 4. Conclusions and Perspectives

Collectively, our work reveals critical residues in Dph1 that likely contact SAM and are functionally relevant for diphtamide synthesis in the model eukaryote *S. cerevisiae* (Figure 5). In addition, our work provides a molecular explanation for the pathological effects of clinically relevant mutations in human DPH1 recently shown to associate with DDS [10,26]. The essential contact residue His-261 in yeast Dph1 (Figure 5) corresponds to His-240 in human DPH1 (Figure 1B and Figure S2), for which the H240R allele has been shown to compromise diphthamide synthesis activity [10]. Moreover, the human L350H variant is a DDS candidate allele [10] and corresponds to Leu-371 in yeast Dph1 (Figure S6), a direct neighbor of Arg-370, which we showed is an essential SAM pocket residue (Figure 5). A similar rationale may apply to human DDS variant P348S (Pro-369 in yeast) [10], which flanks the FeS cluster ligand Cys-447 (Cys-368 in yeast) [8] (Figure S6). Thus, pathogenic L350H and P348S variants likely affect the side chain positioning of Arg-349, the SAM pocket residue in human DPH1 conserved to yeast Arg-370 (Figure 5 and Figure S6).

Guided by the archaeal sructure in complex with the SAM cosubstate, our study thus pinpoints a potential SAM-binding pocket in eukaryal Dph1•Dph2 heterodimers that is structurally similar to archaeal Dph2•Dph2 homodimers but exclusively restricted to one of the two subunits in the yeast RS enzyme: Dph1 (Figure 5). In contrast to archaeal homodimers (Dph2•Dph2), the eukaryal enzyme is made of different subunits (Dph1•Dph2). We showed here that this asymmetry not only reflects the subunit composition but also the exclusive presence of a potential SAM-binding pocket in subunit Dph1 (Figure 5) and not Dph2. Thus, in terms of evolution, the archaeal prototype Dph2 is much more conserved to Dph1 than to Dph2 from any of the model eukaryotes used in our query. Whether this suggests the yeast or human enzymes arose from an ancient *DPH2* gene duplication and diversification event that may explain today’s peculiar heterodimeric structure remains to be elucidated, such as through gene shuffle and complementation studies in budding yeast or other eukaryotic models of interest.

Nonetheless, the uniqueness of a SAM pocket in Dph1 that we showed is essential for the initiation step of diphthamide synthesis urges for further studies into the individual roles played by the FeS clusters in either subunit of the Dph1•Dph2 enzyme [8]. While the FeS cluster in Dph1 operates catalytically in reductive SAM cleavage and ACP radical formation [11,14,41], the role for the metallic cofactor in Dph2 is far from clear. Potentially, it acts in regulation of the heterodimer rather than direct catalysis. Such an option has been spurred by observations that proper electron flow to Dph1•Dph2 from donor proteins (Cbr1, Dph3•Dph8 and Kti11•Kti13) may help control physiological radical reactions and avoid harmful ones [20,45,46,47,48].

With archaeal (Dph2•Dph2) and eukaryotic (Dph1•Dph2) enzymes being able to form ACP radicals, dimer asymmetry obviously is not a prerequisite for the underlying SAM cleavage [11,14,41]. In our hands, it instead implies an FeS cluster geometry in the SAM-binding pocket that facilitates regio-selective SAM cleavage to specifically liberate the ACP (rather than a classical dAdo) radical. A structural comparison between the SAM pockets of *Cmn*Dph2 and the canonical radical SAM enzyme Elp3 from mice [49] clearly suggests differences in SAM pocket residue composition and the overall SAM orientation upon 4Fe-4S binding (Figure S7). The results obtained here propose that pocket residues in proximity to the methionine moiety of SAM are essential (i.e., Gly-238, His-261, Arg-370 and Asp-374 (Figure 5)). Whether these may determine which of the three carbons bonds with the sulfonium in SAM to break and set ACP radicals free [14,41] is important to study, since this may clarify the conceptual differences between classical members of the RS enzyme family and the non-canonical ones for the diphthamide pathway (i.e., yeast Dph1•Dph2 or human DPH1•DPH2) [50,51].

## Data Availability

All data can be found in the manuscript and the Supplementary Materials.

## Supplementary Materials

The following supporting information can be downloaded at https://www.mdpi.com/article/10.3390/biom13111655/s1. Figure S1: Alignment between archaeal *Cmn*Dph2 and eukaryal Dph1 and Dph2 sequences. Figure S2: The SAM pocket from archaeal *Cmn*Dph2 is conserved in eukaryal Dph1 and not Dph2. Figure S3: Dph1 protein expression analysis from wild-type and various mutant strain backgrounds. Figure S4: Detection of unmodified EF2 pools using Western blot analysis. Figure S5: Structural consequences of alanine mutations in the Dph1 SAM pocket. Figure S6: Structural consequences of DDS-related Dph1 mutations flanking the candidate SAM pocket. Figure S7: Structural comparison of SAM pockets between *Cmn*Dph2 and the canonical radical-SAM enzyme Elp3 from *Mus musculus*. Table S1: Yeast strains used and generated in this study. Table S2: Primers used for PCR-based gene engineering and genomic verification.
