# Supplementary material for: DPH1 Gene Mutations Identify a Candidate SAM Pocket in Radical Enzyme Dph1•Dph2 for Diphthamide Synthesis on EF2
Source: Biomolecules. 2023 Nov 16;13(11):1655. doi: 10.3390/biom13111655 (PMC10669111; doi:10.3390/biom13111655)
Supplement: Supplementary file 1 [file biomolecules-13-01655-s001.zip › biomolecules-2706917-supplementary.pdf]

biomolecules

Supplementary Materials

Communication

DPH1 gene mutations identify a candidate SAM pocket in radical enzyme Dph1•Dph2 for diphthamide synthesis on EF2

Koray Ütkür [1], Sarina Schmidt [1], Klaus Mayer [2], Roland Klassen [1], Ulrich Brinkmann [2] and Raffael Schaffrath [2,*]

1 Institut für Biologie, Fachgebiet Mikrobiologie, Universität Kassel, Kassel, Germany
2 Roche Pharma Research and Early Development (pRED), Large Molecule Research, Roche Innovation Center Munich, Penzberg, Germany
* Correspondence: schaffrath@uni-kassel.de

1. Supplementary Tables

Table S1. Yeast strains used and generated in this study.

Strain	Genotype	Source
BY4741	*MAT**a** his3Δ1 leu2Δ0 met15Δ0 ura3Δ0*	Euroscarf *
Y02262	BY4741 but *dph1Δ::kanMX4*	Euroscarf
KU18	BY4741 but *DPH1-(HA)*$_6$*::HIS3MX6*	This study
KU129	BY4741 but *dph1G238A; KlLEU2*	This study
KU328	BY4741 but *dph1G238A-(HA)*$_6$*::HIS3MX6; KlLEU2*	This study
KU285	BY4741 but *dph1H261A; KlLEU2*	[1]
KU329	BY4741 but *dph1H261A-(HA)*$_6$*::HIS3MX6; KlLEU2*	This study
KU287	BY4741 but *dph1Q321A; KlLEU2*	This study
KU330	BY4741 but *dph1Q321A-(HA)*$_6$*::HIS3MX6; KlLEU2*	This study
KU289	BY4741 but *dph1V349A; KlLEU2*	This study
KU331	BY4741 but *dph1V349A-(HA)*$_6$*::HIS3MX6; KlLEU2*	This study
KU291	BY4741 but *dph1R370A; KlLEU2*	This study
KU332	BY4741 but *dph1R370A-(HA)*$_6$*::HIS3MX6; KlLEU2*	This study
KU293	BY4741 but *dph1D374A; KlLEU2*	This study
KU333	BY4741 but *dph1D374A-(HA)*$_6$*::HIS3MX6; KlLEU2*	This study
KU25	BY4741 but *dph1C368S-(HA)*$_6$*::HIS3MX6*	This study
KU123	BY4741 but *dph1Q321A/V349A; ::loxP::*	This study

* http://www.euroscarf.de/index.php?name=News

biomolecules

Table S2. Primers used for PCR-based gene engineering and genomic verification.

Name	Sequence (5′ → 3′)	Usage *
DPH1KOURAF	CTCATGAACTATCTGCTGCGAATTTTAAGGATAATCGGATA<br>GCCAGCTGAAGCTTCGTACGC	ko
DPH13'UTRLEUF	CGTTTTTGACGGCTTGCAGGCGAAACTAAATTGTCTAAAAT<br>TCAAAACCAGCTGAAGCTTCGTACG	smi
DPH13'UTRLEUR	GAATAAAAATAGGCTTGACCAGCAGTGATATCAAGTTAGA<br>AGGCATTGCATAGGCCACTAGTGGATCTG	ko/smi
DPH15'UTRF	GTGATGGTAGATTATAGCAAG	ko-ver
DPH13'UTRR	GGAAATATGCTTGGCAAACTC	ko-ver
DPH1G238AFW	GGGGTGAAGTATTGGCGTGTACTTCTG	sdm
DPH1G238ARV	CTTTCAGAAGTACACGCCAATACTTCAC	sdm
DPH1H261AFW	CGGTGATGGTAGATTTGCTTTGGAGTC	sdm
DPH1H261ARV	TATCATTGCAGACTCCAAAGCAAATCTACC	sdm
DPH1Q321AFW	GGTGCATTAGGTAGAGCAGGTAATTTAAAC	sdm
DPH1Q321ARV	CAGTGTTTAAATTACCTGCTCTACCTAATG	sdm
DPH1V349AFW	GAAAATTATTCTAAGTGAAGCTTTTCCCC	sdm
DPH1V349ARV	GCTTTTGGGGAAAAGCTTCACTTAG	sdm
DPH1R370AFW	CAGGTCGCATGTCCTGCACTGTCCATC	sdm
DPH1R370ARV	CCCAATCGATGGACAGCGTAGGACATGC	sdm
DPH1D374AFW	CCTAGACTGTCCATCGCTTGGGGTTATG	sdm
DPH1D374ARV	GAAGGCATAACCCCAAGCGATGGACAG	sdm
DPH1C368SFW	CAAATTGATGTTTTTGTTCAGGTCGCATCTCCTAGACTGTCC	sdm
DPH1C368SRV	GAAGGCATAACCCCAATCGATGGACAGTCTAGGAGATGCG<br>ACCTG	sdm
DPH1S2	CATATGTAACAGGAAGACAAGTGACAACAAAAACTATTTA<br>AACTAATCGATGAATTCGAGCTCG	tag
DPH1S3	CGAAGCTAAAGGATACGGGCGTGGGGAAACTCCGAAACA<br>TGCGATTGAACGTACGCTGCAGGTCGAC	tag

* Abbreviations used: (i) ko – gene knock-out

(ii) smi – selection marker insertion

(iii) ko-ver – verification of gene knockout

(iv) sdm – site-directed mutagenesis

(v) tag – epitope tagging

2. Supplementary Figures

Figure S1 (for legend, see next page)

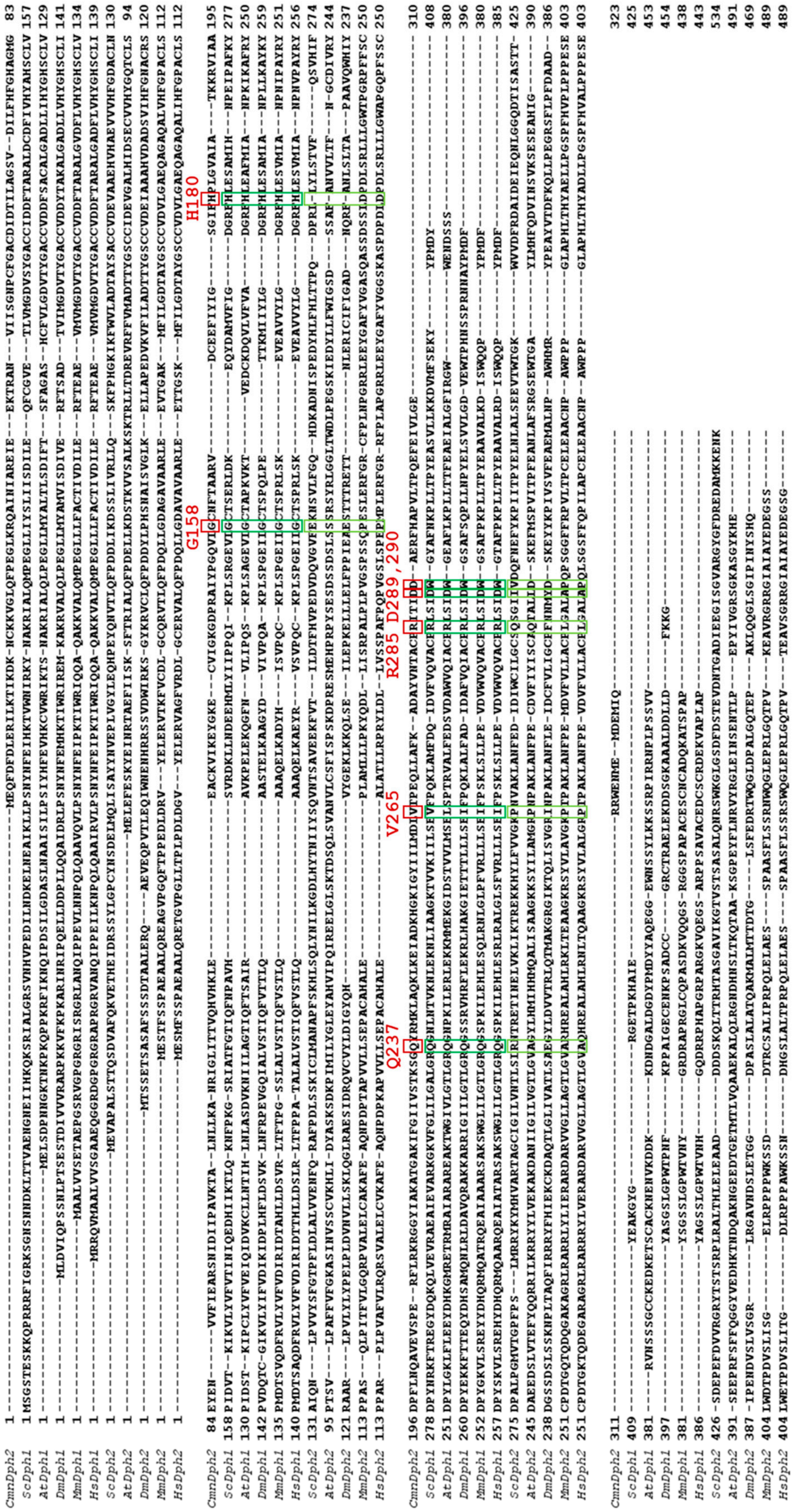

biomolecules

Figure S1. Alignment between archaeal *Cmn*Dph2 and eukaryal Dph1 and Dph2 sequences. Amino acid sequences of *Cmn*Dph2 were aligned to sequences of Dph1 and Dph2 from *S. cerevisiae*, *A. thaliana*, *D. melanogaster*, *M. musculus* and *H. sapiens*. Eukaryal Dph1 residues conserved (dark green) to the SAM pocket residues (G158, H180, Q237, V265, R285 and D289) of *Cmn*Dph2 (red) are shown boxed. The SAM pocket residue in position 290 from *Cmn*Dph2 (D290) is less conserved and occupied in any of the eukaryal Dph1 query sequences with a conserved tryptophan residue. Note that eukaryal Dph2 sequences (light green and boxed) are not that conserved at the sites invariant between Dph1 or *Cmn*Dph2 - except for counterpart analogues to *Cmn*V265 and *Cmn*D290 in Dph2 from *A. thaliana* (D332) and *D. melanogaster* (I309, D335) (see Fig. S2 in bold light green).

Figure S2

*Cmn*Dph2	G158	H180	Q237	V265	R285	D289	D290
*Sc*Dph1	**G238**	**H261**	**Q321**	**V349**	**R370**	**D374**	W375
*At*Dph1	**G208**	**H234**	**Q294**	**L322**	**R344**	**D348**	W349
*Dm*Dph1	**G221**	**H243**	**Q303**	**I331**	**R352**	**D356**	W357
*Mm*Dph1	**G213**	**H235**	**Q295**	**I323**	**R344**	**D348**	W359
*Hs*Dph1	**G218**	**H240**	**Q300**	**I328**	**R349**	**D353**	W354
*Sc*Dph2	E228	-	R315	P343	Q364	I368	V369
*At*Dph2	S195	-	A288	P316	Q337	L348	**D342**
*Dm*Dph2	S197	-	E281	**I309**	F330	Y334	**D335**
*Mm*Dph2	P193	D231	A294	P322	L343	A347	P348
*Hs*Dph2	P193	D231	A294	P322	L343	A347	P348

Figure S2. The SAM pocket from archaeal *Cmn*Dph2 is conserved in eukaryal Dph1, not Dph2. Excerpt of the amino acid sequence alignment (see Fig. S1 above for full details) between eukaryal Dph1 and Dph2 from *S. cerevisiae*, *A. thaliana*, *D. melanogaster*, *M. musculus* and *H. sapiens* and *Cmn*Dph2 (see Fig. S1 for full alignment). Dph1 residues conserved to *Cmn*Dph2 (dark green) and a few scattered analogous Dph2 residues (light green) are highlighted in bold.

Figure S3

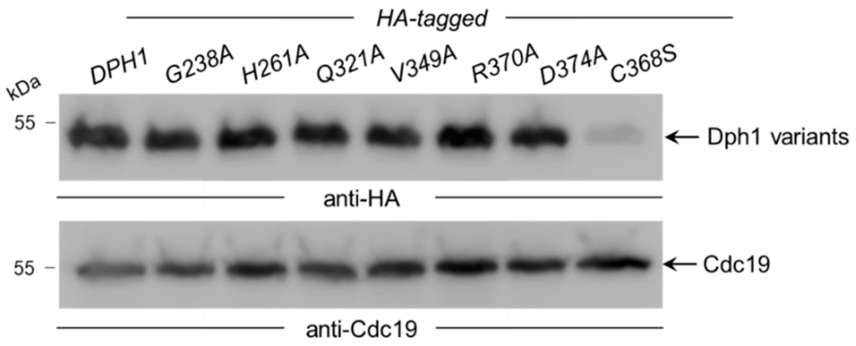

Figure S3. Dph1 protein expression analysis from wild-type and mutant strain backgrounds. Total extracts of yeast strains expressing HA-tagged Dph1 variants as indicated were subjected to Western blot analyses using *anti-HA* and *anti-Cdc19* antibodies to investigate cellular levels of Dph1, and pyruvate kinase Cdc19 as control. As a negative control, HA-tagged *C388S* mutant was included, which produces a highly labile catalytic Dph1 mutant protein.

Figure S4

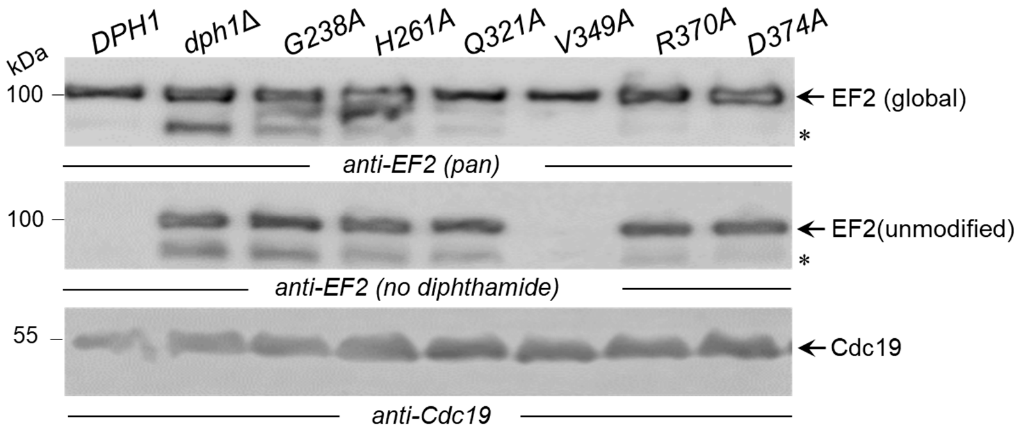

Figure S4. Detection of unmodified EF2 pools using Western blot analysis. Protein extracts of indicated yeast backgrounds were subjected to *anti-EF2(no diphthamide)* Western blots to recognize pools of EF2 not modified with diphthamide [2]. Detection of global EF2 amounts with *anti-EF2(pan)* or Cdc19 with *anti-Cdc19* antibodies is shown in parallel Western blots. EF2 degradation products are marked with an asterisk.

Figure S5

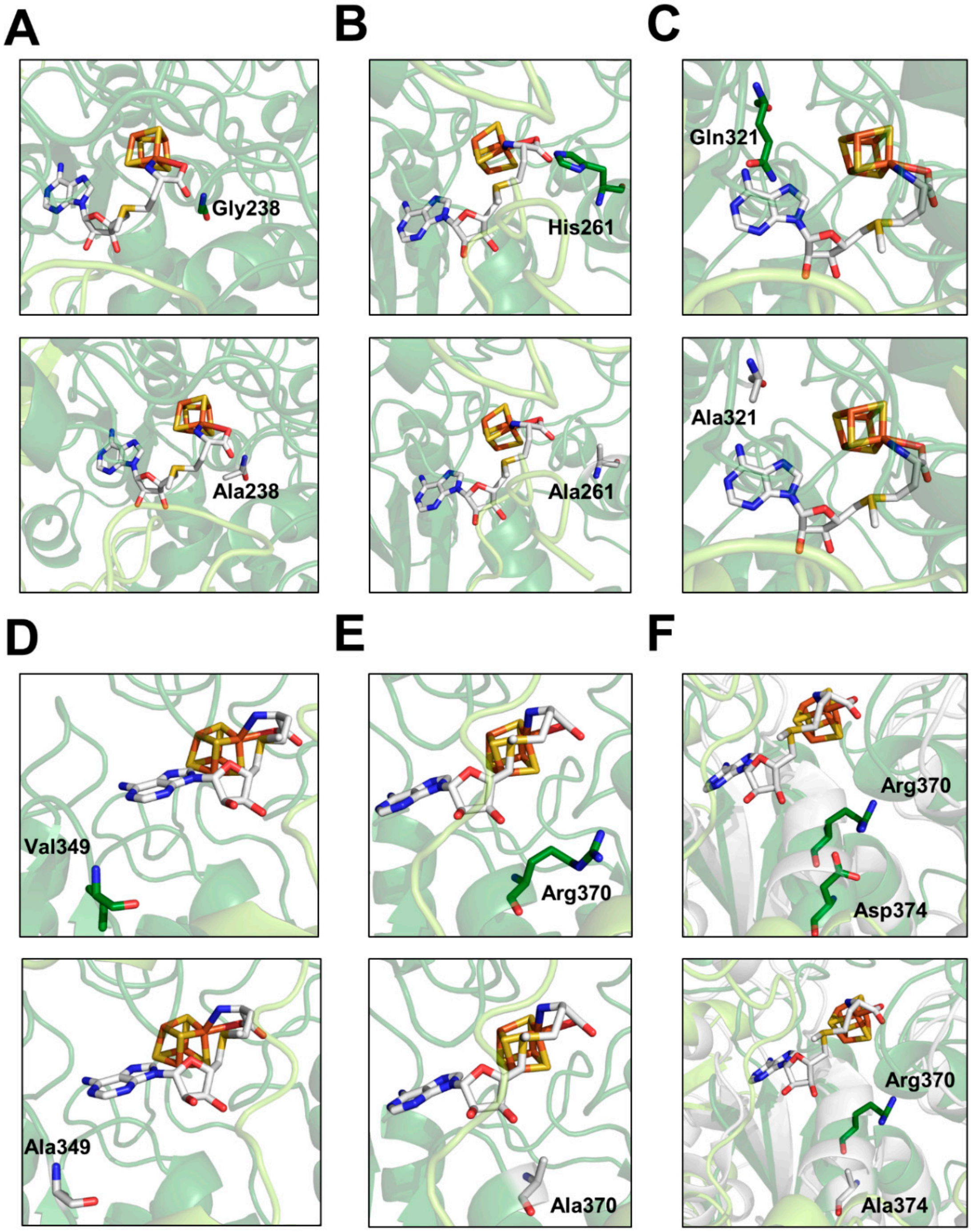

Figure S5. Structural consequences of alanine-mutations in the Dph1 SAM pocket. Alphafold/ColabFold-based [3,4] structural model of Dph1•Dph2 from *S. cerevisiae* was aligned to the structure of *Cmn*Dph2 in complex with SAM (PDB: 6BXN [5]). Protein structures of *Cmn*Dph2 were hidden and cartoon structures of *Sc*Dph1•Dph2 shown with 66% transparency to emphasize stick structures. Individual residues of the candidate SAM pocket (**A**-**F**, upper panels), respectively) were simulated upon alanine substitutions (**A**-**F**, lower panels) and illustrated using PyMOL version 1.3.

Figure S6

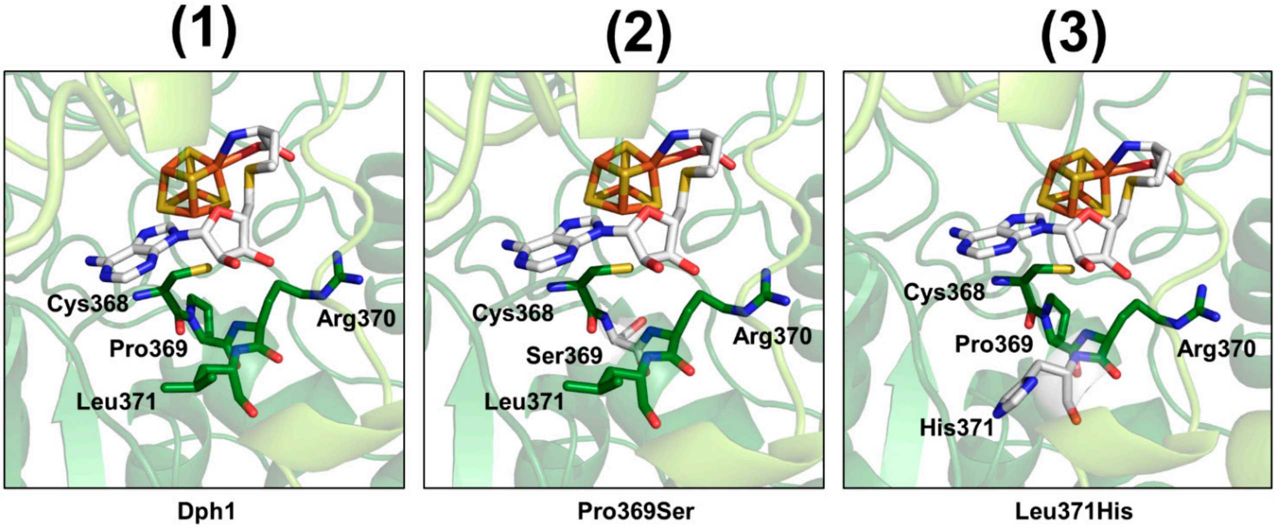

Figure S6. Structural consequences of DDS-related Dph1 mutations flanking the candidate SAM pocket. AlphaFold/ColabFold-based [3,4] structural model of Dph1•Dph2 from *S. cerevisiae* was aligned to the structure of *Cmn*Dph2 in complex with SAM (PDB:6BXN [5]). Protein structures of *Cmn*Dph2 were hidden and cartoon structures of *Sc*Dph1•Dph2 were given with 66% transparency to emphasize stick structures. DDS-related mutations P369S (2) and L371H (3) are illustrated compared to Dph1 wild-type (1) using PyMOL version 1.3.

Figure S7

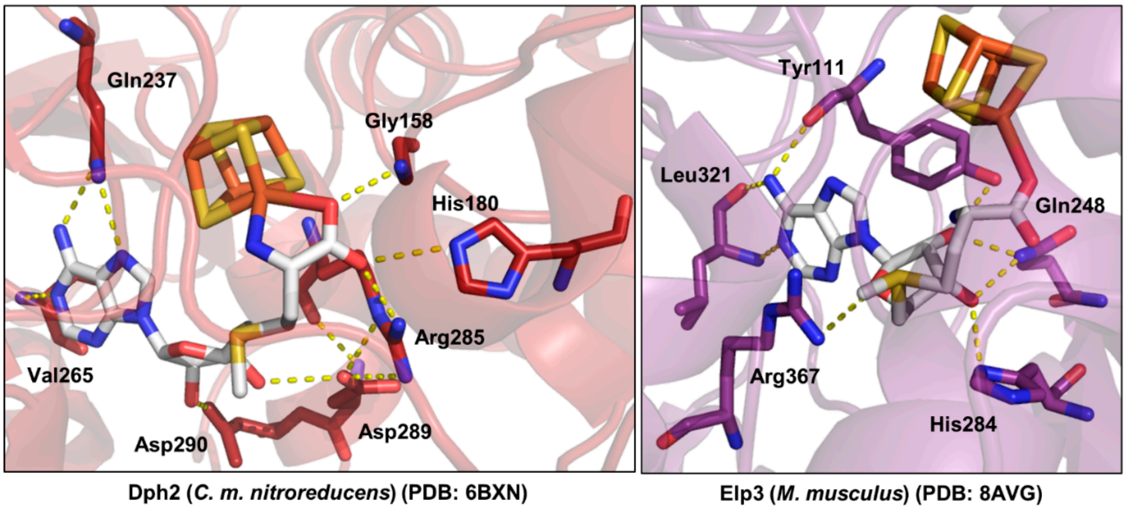

Figure S7. Structural comparison of SAM pockets between *Cmn*Dph2 and the canonical radical-SAM enzyme Elp3 from *Mus musculus*. Illustrations of SAM pockets within the structures of Dph2 from *Candidatus methanoperedens nitroreducens* (PDB:6BXN [5]*)* (left, firebrick red) and Elp3 from *Mus musculus* (PDB: 8AVG [6]) (right, violetpurple) show similarities and differences in comparison. Note that enzymes interact with the respective adenine moiety of SAM using a branched chain amino acid each (Dph2: Val265 and Elp3: Leu321). However, Elp3 occupies the SAM ribose moiety with four interaction partners (Arg367, His284, Gln248 and Tyr111), while Dph2 only pairs the ribose with Asp289 and Asp290 (the latter of which is not conserved in eukaryotes). In Elp3, the C_2 and C_3 OH-groups of the SAM ribose moiety are oriented towards the 4Fe-4S cluster, unlike in Dph2. The respective methionine moieties differ between enzymes as well regarding interaction partner counts and orientation. Structures were illustrated using PyMOL version 1.3.

Original images

Figure S8

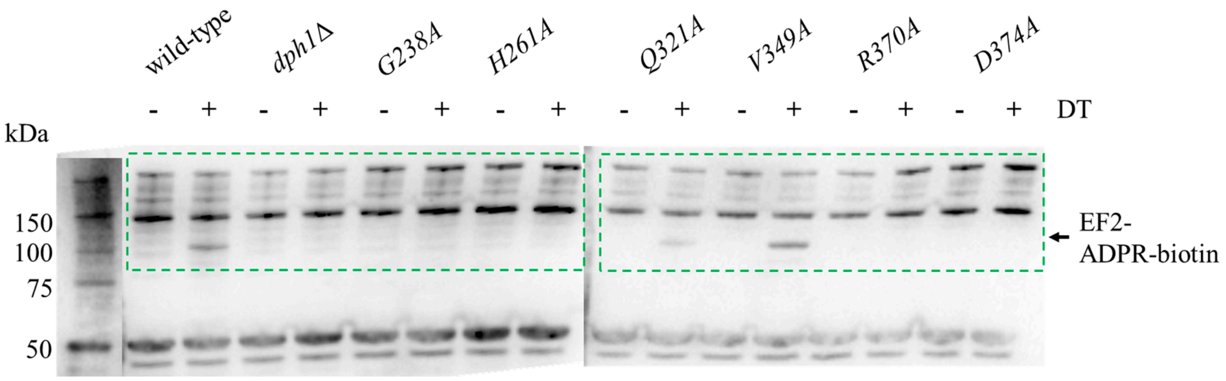

Figure S8. Original uncropped Western blot images underlying the data presented in Figure 3 of the main text (indicated by the areas of the green dotted boxes).

Figure S9

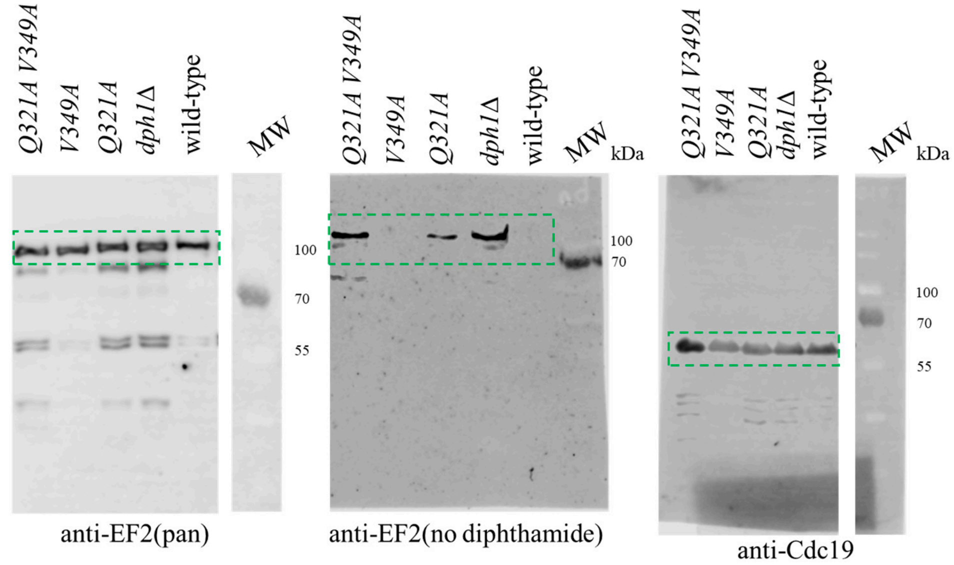

Figure S9. Original, uncropped Western blot images underlying the data presented in Figure 4B of the main text (indicated by the areas of the green dotted boxes).

3. Supplementary References

1. Ütkür K, Mayer K, Khan M, Manivannan T, Schaffrath R, Brinkmann U. DPH1 and DPH2 variants that confer susceptibility to diphthamide deficiency syndrome in human cells and yeast models. Dis. Model. Mech. **2023**, 16, dmm050207, doi: 10.1242/dmm.050207.
2. Stahl S, da Silva Mateus Seidl AR, Ducret A, Kux van Geijtenbeek S, Michel S, Racek T, Birzele F, Haas AK, Rueger R, Gerg M, Niederfellner G, Pastan I, Brinkmann U. Loss of diphthamide pre-activates NF-kappaB and death receptor pathways and renders MCF7 cells hypersensitive to tumor necrosis factor. Proc. Natl. Acad. Sci. USA **2015**, 112, 10732–10737. doi: 10.1073/pnas.1512863112.
3. Jumper J, Evans R, Pritzel A, Green T, Figurnov M, Ronneberger O, Tunyasuvunakool K, Bates R, Žídek A, Potapenko A, Bridgland A, Meyer C, Kohl SAA, Ballard AJ, Cowie A, Romera-Paredes B, Nikolov S, Jain R, Adler J, Back T, Petersen S, Reiman D, Clancy E, Zielinski M, Steinegger M, Pacholska M, Berghammer T, Bodenstein S, Silver D, Vinyals O, Senior AW, Kavukcuoglu K, Kohli P, Hassabis D. Highly accurate protein structure prediction with AlphaFold. Nature **2021**, 596, 583-589. doi: 10.1038/s41586-021-03819-2.
4. Mirdita M, Schütze K, Moriwaki Y, Heo L, Ovchinnikov S, Steinegger M. ColabFold: making protein folding accessible to all. Nat. Methods **2022**, 19, 679-682. doi: 10.1038/s41592-022-01488-1.
5. Dong M, Kathiresan V, Fenwick MK, Torelli AT, Zhang Y, Caranto JD, Dzikovski B, Sharma A, Lancaster KM, Freed JH, Ealick SE, Hoffman BM, Lin H. Organometallic and radical intermediates reveal mechanism of diphthamide biosynthesis. Science **2018**, 359, 1247-1250. doi: 10.1126/science.aao6595.
6. Jaciuk M, Scherf D, Kaszuba K, Gaik M, Rau A, Kościelniak A, Krutyhołowa R, Rawski M, Indyka P, Graziadei A, Chramiec-Głąbik A, Biela A, Dobosz D, Lin TY, Abbassi NE, Hammermeister A, Rappsilber J, Kosinski J, Schaffrath R, Glatt S. Cryo-EM structure of the fully assembled Elongator complex. Nucleic Acids Res. **2023**, 51, 2011-2032. doi: 10.1093/nar/gkac1232.
